# Economic evaluation of digital health interventions to improve quality-adjusted life years in adolescents living with HIV in Ethiopia

**DOI:** 10.3389/fpubh.2026.1718940

**Published:** 2026-01-23

**Authors:** Abayneh Tunje, Pia Lundqvist, Magnus C. Persson, Guðrún Kristjánsdóttir, Rúnar Vilhjálmsson, Degu Jerene

**Affiliations:** 1 Department of Health Sciences, Faculty of Medicine, Lund University, Lund, Sweden; 2 School of Public Health, College of Medicine and Health Sciences, Arba Minch University, Arba Minch, Ethiopia; 3 Faculty of Nursing and Midwifery, School of Health Sciences, University of Iceland and the Landspitali University Hospital, Reykjavík, Iceland; 4 KNCV Tuberculosis Foundation, The Hague, Netherlands

**Keywords:** adolescents, antiretroviral therapy, cost-effectiveness, EQ-5D-3L, HIV, QALYs, quality-adjusted life years, SMS

## Abstract

**Introduction:**

The rapid expansion of mobile phone access has enabled low-cost SMS interventions to support adherence to antiretroviral therapy (ART). Although text messaging improves adherence and retention in HIV care, its cost-effectiveness for enhancing quality of life remains uncertain, particularly among adolescents in resource-limited settings. This study evaluated whether SMS reminders improve retention in care and quality-adjusted life years (QALYs) among adolescents living with HIV in Ethiopia. We hypothesized that SMS reminders would increase adherence and retention, leading to measurable gains in quality of life.

**Methods:**

A 6-month economic evaluation was conducted from the health system perspective, comparing daily SMS reminders plus standard care vs. standard care alone. HRQoL was measured using the EuroQol EQ-5D-3L instrument, which includes five domains: mobility, self-care, usual activity, pain/discomfort, and anxiety/depression. Utility values were derived using the Zimbabwean EQ-5D-3L value set as a proxy for Ethiopia and converted into QALYs. Costs were categorized as fixed (planning, system development, training) and variable (personnel, SMS communication, equipment, and overhead), valued in 2023 US dollars (USD; 1 USD = 54.7442 ETB). The Incremental Cost-Effectiveness Ratio (ICER) was calculated as ΔCost/ΔQALY. The WHO-CHOICE cost-effectiveness threshold (<3 × GDP per capita; $3,636 for Ethiopia, 2023) guided interpretation.

**Results:**

Daily SMS reminders were cost-effective according to WHO thresholds, with an ICER of $1,037.00 per QALY gained. Accounting for potential retention improvements, the ICER decreased to $864.00 per QALY gained. Intervention participants had higher utility scores (0.9515 vs. 0.9030) and QALYs (0.4760 vs. 0.4510) compared to controls.

**Discussion:**

SMS-based adherence interventions offer a cost-efficient approach to improving ART outcomes by enhancing adherence and retention. Anxiety/depression and pain/discomfort were key determinants of QALYs. Future research should assess long-term cost-effectiveness and scalability in resource-limited settings.

**Trial Registration:**

PACTR202107638293593.

## Introduction

Quality of life (QoL) refers to an individual's overall physical, psychological, and social wellbeing as perceived within the context of their health condition (1). In health economics, QoL is often expressed through the concept of quality-adjusted life years (QALYs), which combine both the quantity and quality of life lived into a single standardized measure (2). QALYs are widely recognized as the principal metric in economic evaluations because they enable comparisons across different interventions and disease conditions, supporting evidence-informed decision-making in health policy (3). Unlike clinical outcomes that primarily reflect physiological or symptomatic improvements, QALYs incorporate patient-centered perspectives and health-related wellbeing, offering a more comprehensive evaluation of intervention benefits (4).

Adolescents living with HIV (ALHIV) experience multiple challenges such as stigma, treatment fatigue, limited autonomy, and psychosocial stress that negatively impact adherence to antiretroviral therapy (ART) and, consequently, their QoL and treatment outcomes (5, 6). Although ART has transformed HIV infection into a manageable chronic condition, allowing individuals to achieve near-normal life expectancy (7), maintaining adherence remains a persistent problem among adolescents, particularly in sub-Saharan Africa (SSA) (8, 9).

Mobile health (mHealth) interventions, such as SMS reminders and mobile app–based adherence support, have been increasingly applied to enhance treatment adherence and health outcomes in chronic diseases, including HIV (10). Evidence from global systematic reviews demonstrates that mHealth interventions can significantly improve ART adherence and viral suppression rates (11, 12). In low- and middle-income countries (LMICs), and particularly in SSA, studies have shown that SMS-based reminders are effective in supporting medication adherence and retention in care among adults living with HIV (13–15). However, the evidence for their impact among adolescents remains limited, and few studies have examined their cost-effectiveness (16, 17).

Ethiopia has achieved substantial ART scale-up over the past two decades, rising from around 4% coverage in 2005/6 to nearly 98% of diagnosed people living with HIV on treatment by 2022 (9). Despite this progress, adolescents aged 15–19 years continue to lag behind adults in ART initiation, adherence, and viral suppression (18, 19), highlighting a critical gap that must be addressed to meet national and global HIV targets. Moreover, the integration of QALY-based evaluation within adolescent HIV research is scarce, limiting understanding of the broader health and economic benefits of digital adherence support. Therefore, this study aimed to evaluate the effect of an SMS-based adherence intervention on QALYs among adolescents living with HIV in Ethiopia.

## Methods and materials

### Setting and study period

The study was conducted from 5 July 2022 to 28 February 2023 in six hospitals and five health centers providing HIV care to adolescents across five zones of South Ethiopia. Hospitals with comprehensive adolescent HIV services were purposively selected, and all eligible facilities were included to ensure complete coverage.

### Study population

All eligible adolescents in both the intervention and control arms (*n* = 306, 1:1 ratio) were provided with mobile phones to ensure uniform access to communication with healthcare providers. However, only participants in the intervention arm received SMS reminders as part of the adherence support intervention.

Control arm participants received only standard care per national guidelines (20), including clinical assessment, index case testing, mental health and nutritional support, sexual and reproductive health, psychosocial support, community care, youth-friendly services, and transition to adult care. Additionally, all adolescents received a mobile phone for communication with healthcare providers. Follow-up data and blood samples were collected at each visit.

### Ethics statement

The study was conducted in accordance with the Declaration of Helsinki and received ethical approval from the Swedish Regional Ethical Review Board (Reg. no. 2019-03433), the National Research Ethics Review Committee, Ethiopia (MoSHE/RD/142/2869/20), and the Institutional Research Ethics Review Board at Arba Minch University (IRB-113/11). Permission to collect data was obtained from the administration of each participating hospital and health center. For participants aged 10–17 years, written informed consent was obtained from parents or guardians, along with assent from the adolescents, including permission to access their medical records. Participants aged ≥18 years provided written informed consent themselves.

### Socio-demographic and healthcare-related characteristics

Data on socio-demographic and healthcare-related characteristics were collected to describe the study population and account for potential confounders. Socio-demographic variables included age, sex, residence (urban/rural), education, marital status, and living arrangement (with family or alone). Healthcare-related variables comprised type of health facility (hospital or health center), duration on ART, and mode of ART delivery. Information was obtained using a structured questionnaire and verified against medical records. Data collection occurred at baseline (Visit 1), midline at 3 months (Visit 2), and endline at 6 months (Visit 3) using the mobile version of REDCap (21).

### Health outcomes measurement

Health-related quality of life (HRQoL) was measured using the EuroQol EQ-5D-3L instrument (22), which includes five dimensions: mobility, self-care, usual activities, pain/discomfort, and anxiety/depression. In contrast, the EQ-5D-5L lacks local validation and established value sets in Ethiopia, which limits comparability and interoperability for economic evaluation in this context.

Participants rated their health status in each domain on three levels: no problems, some problems, or extreme problems. For analysis, responses were dichotomized as “no problem” (0) and “has problems” (1), with levels 2 and 3 recoded as “has problems” to represent functionality status. These domains are used to derive utility values for QALY calculation. Responses obtained from the EQ-5D-3L instrument were changed into an index value, which in turn was converted to QALY or utility scores using country-specific value sets. Since an Ethiopian EQ-5D tariff has not yet been developed, we used the Zimbabwean EQ-5D-3L value set that has been widely used in sub-Saharan Africa due to its similar cultural and socio-economic context (22). The index values were converted into utility scores by applying the standard EQ-5D valuation methodology, where each health state described by the five EQ-5D dimensions is assigned a weight derived from population preferences elicited through TTO techniques (23, 24). Utility values range from 1 (perfect health) to 0 (death), whereas utility scores below 0 represent health states considered worse than death (25).

### Cost categories

We classified cost data into fixed and variable costs. Fixed costs encompassed activities such as intervention planning, site preparation, development of the mobile SMS text messaging management system, and initial training. Variable costs covered recurring expenses necessary to maintain the intervention, categorized into distinct input groups: personnel, communication, equipment, and overhead. Personnel costs included salaries, benefits, and allowances for facility-based health workers, as well as staff responsible for supervision and coordination. Communication costs encompassed cloud hosting of solution platform fees, internet data bundles for the Mobile system, and SMS messaging expenses. Equipment included mobile phones, desktops, and routers. All costs were converted to 2023 US dollars using the average ETB–USD exchange rate (1 USD = 54.7442 ETB; National Bank of Ethiopia, 2023) to reflect the economic context closest to the study period and enable comparison with the WHO-CHOICE cost-effectiveness threshold (26). Research-related expenses, including research time as well as supervisors' travel and accommodation costs, were excluded to ensure the analysis reflected the actual program costs when implemented and scaled locally.

### Data analysis

Data were compiled and analyzed using Microsoft Excel 2019 (Microsoft Office 365) to estimate total and incremental costs, as well as incremental cost-effectiveness ratios (ICERs). Study data were initially collected in REDCap and subsequently exported to Excel for analysis. Two trained research assistants independently entered all data and performed double-checks to ensure accuracy. Comparisons between the intervention and control groups were conducted using appropriate statistical tests. All tests were two-sided with a significance level of 0.05, and 95% confidence intervals (CIs) were reported to quantify the precision of estimates, including differences in QALYs, utility scores, and adherence outcomes.

ICERs were calculated as the ratio of incremental cost to incremental QALYs gained:


$$\begin{array}{l} ICER=\frac{QALYs\;SMS\;-\;QALYs\;Standard\;care}{Cost\;SMS\;-\;Cost\;Standard\;care} \end{array}$$

Costs were assessed from the health system perspective and categorized as fixed (planning, system development, training, equipment) and variable (personnel, SMS communication, office supplies, overhead). Resource use was measured based on actual data, with each unit multiplied by its respective cost (Supplementary Table S1). All costs were converted to 2023 US dollars using the average ETB–USD exchange rate (1 USD = 54.7442 ETB; National Bank of Ethiopia, 2023). The incremental cost represents the additional resources required to deliver the SMS intervention compared to standard care.

## Results

### Socio-demographic and healthcare-related characteristics

A total of 306 adolescents living with HIV were included in the analysis, with equal distribution between the intervention group (*n* = 153) and the control group (*n* = 153). Most participants (61.4%) were aged 15–19 years (intervention: 60.8%; control: 62.1%), and 56.2% were male (intervention: 50.9%; control: 61.4%). The majority resided in urban areas (73.2%) and were students (94.4%). Regarding healthcare access, 248 participants (81%) received services from hospitals, while the remaining 19% attended health centers. Nearly all adolescents (97.1%) were unmarried, and most (93.8%) lived with their family; only a small proportion lived alone (6.2%). These distributions were similar across both study arms.

### Functionality status by EQ-5D-3L dimensions

**Figure 1:**
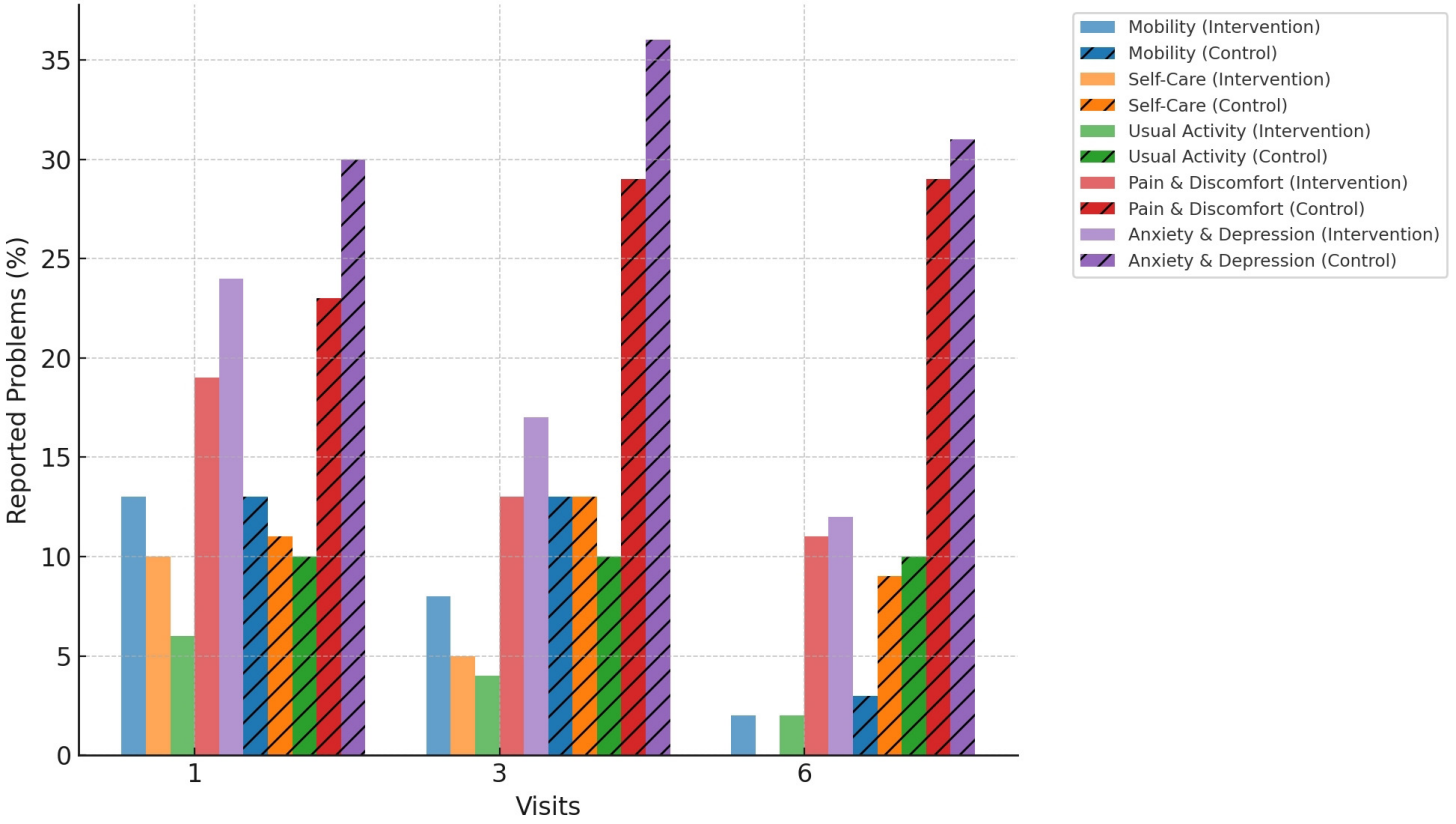
Health problems reported in EQ-5D health dimensions among adolescents living with HIV in South Ethiopia, 2024.

The proportion of adolescents reporting problems in each EQ-5D-3L dimension is presented in Figure 1. At Visit 2, 29% of participants in the control group reported problems with Pain/Discomfort, compared to 18% in the intervention group (χ^2^ = 4.65, df = 1, *p* = 0.031), indicating a higher proportion of reported problems in the control arm. No significant differences were observed in the other dimensions at this time point (all *p* > 0.05).

Over the 6-month period, the intervention group demonstrated improvements in self-care, usual activity, and anxiety/depression compared to the control group. Anxiety/depression decreased from 24% at Visit 1 to 12% at Visit 3 in the intervention group, whereas the control group showed a slight increase from 30 to 31% (χ^2^ = 6.28, df = 1, *p* = 0.012). These findings indicate that adolescents in the intervention group reported fewer problems in key EQ-5D-3L dimensions over the study period, which are dimensions relevant to health-related quality of life and QALYs.

### EQ-5D-3L utility scores

Mean utility scores and QALYs for each study arm are summarized in Table 1. Over 6 months, participants in the intervention group had higher mean utility scores compared to the control group (0.9206 ± 0.2010 vs. 0.8963 ± 0.2400; *t* = 2.48, df = 914, *p* = 0.013). The mean QALYs were also higher in the intervention group (0.4760 ± 0.1040) than in the control group (0.4510 ± 0.1200), with a mean difference of 0.0250 (95% CI: 0.0123–0.0363), reflecting a clinically meaningful improvement consistent with the Minimal Clinically Important Difference (MCID) for EQ-5D-3L (0.03–0.07) (27).

**Table 1:** EQ-5D-3L utility scores of adolescents living with HIV, South Ethiopia, 2023.

**Time of measurement**	**Intervention arm (*****n*** = **153)**		**Control arm (*****n*** = **153)**	
	**Mean utilities (SD)**	**Mean QALY (SD)**	**Mean utilities (SD)**	**Mean QALYs (SD)**
Baseline (visit 1)	0.9222 (0.20)	0.4611 (0.10)	0.9031 (0.22)	0.4515 (0.11)*
3rd month (visit 2)	0.9274 (0.18)	0.4637 (0.09)	0.8903 (0.24)	0.4451 (0.12)
6th month (visit 3)	0.9122 (0.22)	0.4561 (0.11)*	0 0.8955 (0.25)	0.4478 (0.12)
Overall	0.9206 (0.20)	0.4603 (0.10)	0.8963 (0.24)	0.4481 (0.12)

SD, standard deviation.

* n = 152 due to one participant lost to follow-up at 6 months.

^**^n = 151 due to two participant lost to follow-up at baseline.

### Missing data

Three participants in the intervention group and two in the control group were lost to follow-up by Month 6. Analyses were conducted using complete cases; sensitivity analyses including last observation carried forward yielded consistent results (Table 2).

**Table 2:** Quality-adjusted life years of adolescents living with HIV over a 6-month follow-up, South Ethiopia, 2023.

**Study arm**	**Observed**	**Mean**	**Std.err**	**Std.dev**.	**95% CI**	
Intervention arm	458	0.475	0.003	0.070	0.469	0.482
Standard of care arm	457	0.451	0.005	0.108	0.441	0.461
Difference		0.024	0.006		0.012	0.036

Std. err., standard error; Std. dev., standard deviation; 95% CI, 95% confidence interval.

The mean QALYs for adolescents over the present study's 6-month period was 0.4637, with a standard error of 0.02. The lower QALY score may suggest a decrease in life expectancy, a lower quality of life, or a mix of both. Adolescents who received reminder text messages had a mean QALY of 0.476 with a standard error of 0.003, compared to 0.451 with a standard error of 0.005 in the control group. Adolescents living with HIV often experience reduced QALY scores due to challenges such as chronic disease management, stigma, and psychosocial issues.

## Discussion

This study is the first to provide empirical evidence on health state utility values among adolescents living with HIV in Ethiopia using the EQ-5D-3L instrument. The findings demonstrate that adolescents in the intervention arm experienced notable improvements in self-care, usual activities, and anxiety/depression compared to the standard-of-care group. The observed improvement in mental health among adolescents in the intervention arm is consistent with previous studies showing that integrated psychosocial support can reduce anxiety and depressive symptoms. Additionally, improvements were observed in functional domains, with a substantial reduction in self-care difficulties and a modest decline in limitations in usual activities, suggesting broader benefits of the intervention on daily functioning over time. These benefits are likely mediated through improved self-efficacy, reduced treatment-related stress, and strengthened patient–provider communication (6, 28).

The mean utility score was higher in the intervention group (0.9206, SD: 0.201) than in the control group (0.8963, SD: 0.240), indicating a clinically meaningful difference in perceived health status. Adolescents in the intervention group experienced an average improvement of 4.8 percentage points in HRQoL, reflecting the intervention's positive effect. These results are consistent with studies from SSA showing that supportive counseling and digital health interventions improve adherence and mental wellbeing among adolescents living with HIV (11, 15, 22, 29, 30). A study in Kenya reported that structured psychosocial support significantly improved EQ-5D-based HRQoL scores among youth receiving ART (31). Similarly, research in South Africa and Uganda found that reducing anxiety and depression symptoms was associated with improved adherence and viral suppression, which translated into higher QALYs (32–34).

Our findings are consistent with earlier evidence showing that depression and anxiety are key determinants of HRQoL among people living with HIV (35). Improvements in psychological wellbeing have important implications beyond clinical outcomes, as mental health is a key contributor to health-related quality of life and QALY gains. The observed reduction in anxiety and depression may therefore partly explain the higher utility scores and QALYs observed in the intervention arm.

The study has several strengths. It was conducted across multiple hospitals and health centers in both urban and rural settings and included a relatively large cohort of adolescents. To our knowledge, this is the first study in Ethiopia to estimate EQ-5D-3L utility values among adolescents living with HIV, providing a baseline reference for future cost-utility analyses and intervention evaluations. However, some limitations should be acknowledged. The EQ-5D-3L may have limited sensitivity to small changes in HRQoL, and self-reported data may be subject to social desirability bias. Additionally, the absence of a locally derived value set may slightly affect utility precision.

## Conclusion

This study evaluated the effect of SMS-based adherence support on quality-adjusted life years (QALYs) among adolescents living with HIV in Ethiopia. The intervention significantly improved HRQoL, particularly by reducing anxiety and depression and enhancing self-care and daily functioning, compared to standard care. These findings demonstrate that low-cost digital adherence interventions can effectively improve both mental health and overall health outcomes in this population. The utility values derived provide a valuable reference for future economic evaluations and support the integration of psychosocially informed digital interventions into adolescent HIV care in resource-limited settings.

## Data Availability

The raw data supporting the conclusions of this article will be made available by the authors, without undue reservation.
