# Supplementary material for: Economic evaluation of digital health interventions to improve quality-adjusted life years in adolescents living with HIV in Ethiopia
Source: Front Public Health. 2026 Jan 23;14:1718940. doi: 10.3389/fpubh.2026.1718940 (PMC12876147; doi:10.3389/fpubh.2026.1718940)
Supplement: Supplementary file 1 [file Table_1.docx]

**Supplementary Table S1. Intervention Costs per Participant in Ethiopia (USD, 2023)**

| Intervention Activity | Total Cost (ETB) | Total Cost (USD) | Cost per Individual (ETB) | Cost per Individual (USD) |
| --- | --- | --- | --- | --- |
| Personnel |  |  |  |  |
| Local service provider IT support | 24,895 | 455.00 | 162.79 | 2.97 |
| Server administrator | 29,893 | 546.00 | 195.37 | 3.57 |
| Communication |  |  |  |  |
| Cloud hosting of solution | 37,347 | 682.20 | 244.00 | 4.46 |
| Message provider | 14,950 | 273.00 | 97.72 | 1.78 |
| Internet service cost | 24,895 | 455.00 | 162.79 | 2.97 |
| Equipment |  |  |  |  |
| Mobile device for 153 | 278,923 | 5,094.09 | 1,823.92 | 33.30 |
| Server | 29,893 | 546.00 | 195.37 | 3.57 |
| Reuter | 24,895 | 455.00 | 162.79 | 2.97 |
| Total | 465,592 | 8,506.29 | 3,043.25 | 55.60 |
